# Zika Virus Epidemic in Pregnant Women, Dominican Republic, 2016–2017 

**DOI:** 10.3201/eid2502.181054

**Published:** 2019-02

**Authors:** Farah Peña, Raquel Pimentel, Shaveta Khosla, Supriya D. Mehta, Maximo O. Brito

**Affiliations:** Ministry of Health, Santo Domingo, Dominican Republic (F. Peña, R. Pimentel);; University of Illinois at Chicago, Chicago, Illinois, USA (S. Khosla, S.D. Mehta, M.O. Brito)

**Keywords:** Zika virus, arbovirus, Dominican Republic, pregnancy, microcephaly, viruses

## Abstract

Zika virus infection during pregnancy may result in birth defects and pregnancy complications. We describe the Zika virus outbreak in pregnant women in the Dominican Republic during 2016–2017. We conducted multinomial logistic regression to identify factors associated with fetal losses and preterm birth. The Ministry of Health identified 1,282 pregnant women with suspected Zika virus infection, a substantial proportion during their first trimester. Fetal loss was reported for ≈10% of the reported pregnancies, and 3 cases of fetal microcephaly were reported. Women infected during the first trimester were more likely to have early fetal loss (adjusted odds ratio 5.9, 95% CI 3.5–10.0). Experiencing fever during infection was associated with increased odds of premature birth (adjusted odds ratio 1.65, 95% CI 1.03–2.65). There was widespread morbidity during the epidemic. Our findings strengthen the evidence for a broad range of adverse pregnancy outcomes resulting from Zika virus infection.

## Abstract 2

During the epidemic, almost 10% of pregnancies in acute infection resulted in fetal loss; 3 cases of fetal microcephaly were reported.

Zika virus is a flavivirus transmitted by the bite of the *Aedes* mosquito (*1*), horizontally through sexual transmission (*2*–*4*), and vertically during pregnancy and delivery (*5*). Most persons infected with Zika virus are asymptomatic or experience a relatively mild self-limited illness characterized by fever, conjunctivitis, arthralgia, and rash. In adults, Zika virus infection has been associated with Guillain-Barré Syndrome (GBS) and meningoencephalitis (*6*–*8*). When acquired during pregnancy, however, the infection has been linked to fetal microcephaly, intrauterine growth retardation, and ophthalmologic abnormalities in the infant (*9*–*12*). There is evidence that fetal neurologic abnormalities are most severe when infection occurs in early pregnancy, during embryological development of the central nervous system (*13*).

The current Zika virus pandemic began in French Polynesia and in Yap Island, Federated States of Micronesia, in 2013 (*14*,*15*). An explosive outbreak began in the Americas in 2014 with a cluster of cases reported in Easter Island, Chile (*16*), eventually moving to northeastern Brazil, where a large number of cases occurred over the span of 1 year (*17*,*18*). Subsequently, the epidemic progressed to the north of South America and to the Caribbean basin (*19*,*20*).

The Ministry of Health (MoH) of the Dominican Republic instituted epidemiologic surveillance for Zika infection in December 2015 in preparation for the possible introduction of the virus. In January 2016, serum samples of suspected case-patients were sent to the US Centers for Disease Control and Prevention (CDC), which assisted in confirming the first 10 cases of Zika virus infection in the provinces of Santo Domingo, Jimaní, and Barahona (*21*). By end of April 2017, >5,000 cases (suspected and confirmed) had been reported in 28/32 country provinces (*22*). Considering the public health implications of Zika virus acquired during pregnancy, we sought to describe the characteristics of the outbreak among pregnant women and to analyze outcomes of pregnancy for women reported to the Dominican Republic MoH during the surveillance period.

## Materials and Methods

### Study Design and Setting

We conducted a cross-sectional analysis of suspected Zika infections among pregnant women reported to the Dominican Republic MoH during the countrywide outbreak, January 2016–April 2017. The Dominican Republic, with a population of nearly 9.5 million, occupies the eastern two thirds of the Caribbean island of Hispaniola (*23*). The Dominican healthcare system is administratively divided in 9 healthcare regions (0–VIII), which include the country’s 31 provinces, 155 municipalities, and the capital city of Santo Domingo (*24*). This study was approved by the Institutional Review Board of the University of Illinois at Chicago (Chicago, IL, USA).

### Surveillance

Epidemiologic surveillance for Zika virus was instituted in December 2015. We trained Dominican Republic MoH personnel on surveillance methods and disseminated public information on the disease and its complications. The MoH assembled a multidisciplinary team of epidemiologists, entomologists, and clinicians from the Epidemiology Directorate and the National Center for the Control of Tropical Diseases to assess the countrywide risk and identify the communities most vulnerable to the spread of the disease. The MoH conducted rapid surveys of syndromic symptomatology in areas suspected to have persons infected with Zika virus (e.g., by informal communications from healthcare providers and local municipalities) to confirm circulation of the virus. In April 2016, the MoH mandated reporting of all suspected cases of GBS, microcephaly, and other congenital abnormalities that might be related to Zika infection. To support this, the MoH introduced a single reporting form for individual cases that was completed by all public and private health centers countrywide with suspected cases. Healthcare facilities transmitted the data via the National System of Epidemiologic Surveillance (SINAVE), the online platform for individual case and outbreak reporting of the MoH.

### Case Definition

We used criteria from the Pan American Health Organization (*25*) to classify cases reported during January 2016–April 2017. Suspected cases were defined as illness in patients with acute onset of rash, fever (>38.2°C), or both and >1 of the following: arthralgia or myalgia, nonpurulent conjunctivitis or conjunctival hyperemia, and headaches not explained by other medical conditions. Probable cases were suspected cases with positive results for Zika virus IgM and no evidence of other arboviral diseases. Confirmed cases were suspected cases with Zika virus RNA detected in urine or blood. Microcephaly was defined as head circumference <2 SD below the mean, adjusted for gestational age and sex, 24 h after birth. Trained MoH staff followed published CDC procedures for measuring head circumference in infants (*26*).

### Laboratory Testing

Testing was performed at CDC’s laboratories in San Juan, Puerto Rico, or at the Dr. Defilló National Public Health Laboratory in Santo Domingo. Laboratory personnel tested available blood or urine specimens with reverse transcription PCR with primers to detect Zika virus RNA. Serum PCR was obtained for patients who sought care within 5 days of symptom onset, whereas urine PCR was obtained for patients who sought care 5–15 days after symptom onset. Samples were discarded if their collection or transportation did not follow the appropriate protocol (i.e., inadequate refrigeration, incorrect sample labeling, insufficient quantity). All women with discarded samples were included in the final analysis as suspected cases.

### Data Collection

The standardized case report form for suspected Zika cases included information on age, sex, pregnancy status, insurance status, place of residence, care setting, signs/symptoms, comorbidities, fetal vital status, and pregnancy or fetal complications. We exported the SINAVE database to a spreadsheet in Excel (Microsoft, http://www.microsoft.com) for daily and weekly analyses and drafted weekly epidemiologic bulletins that were made available on the website of the Epidemiology Directorate of the MoH (www.digepisalud.gob.do). We contacted women to confirm pregnancy outcomes.

### Study Variables

For this analysis, we classified newborns weighing <2,500 g at birth as low birth weight for a full-term newborn. We dichotomized maternal age as <30 years and >30 years for the multivariate analysis. We chose to dichotomize at 30 years instead of 35 years because there were relatively few women >35 years of age. We dichotomized region of residence as Greater Santo Domingo, which included the capital city of Santo Domingo and its suburbs, or others. We categorized insurance status as having any insurance or no insurance. We categorized care setting as hospitalized or nonhospitalized (outpatient medical care and at-home care). We categorized gestational age at the time of maternal Zika virus infection as <12 weeks or >12 weeks. Birth was either premature (<37 weeks) or full term (>37 weeks). We grouped miscarriages and intrauterine fetal demises (IUFD) and categorized them as fetal loss for the multivariable analysis.

### Statistical Analysis

We downloaded data from SINAVE to Excel and imported into SAS version 9.3 (http://www.sas.com) for analyses. We conducted univariate analysis, generated frequencies for categorical variables, and calculated measures of central tendency for continuous variables. We generated an epidemic curve for 2016–2017 by epidemiologic week. We compared distributions of demographic and clinical findings by pregnancy outcome and used the χ^2^ test to obtain p values. We conducted multinomial logistic regression to identify factors associated with early fetal losses (miscarriages and IUFD) and to compare preterm live birth with term live birth. We conducted multivariable analyses using variables that were significant at p<0.20 in unadjusted analyses. Final models retained variables with p<0.10 except for age, which was kept in all modeling due to its potential influence on pregnancy complications. We geomapped cases and created the map in ArcGIS version 10.4.1 (http://www.arcgis.com).

## Results

### Characteristics of Pregnant Women

**Figure 1:**
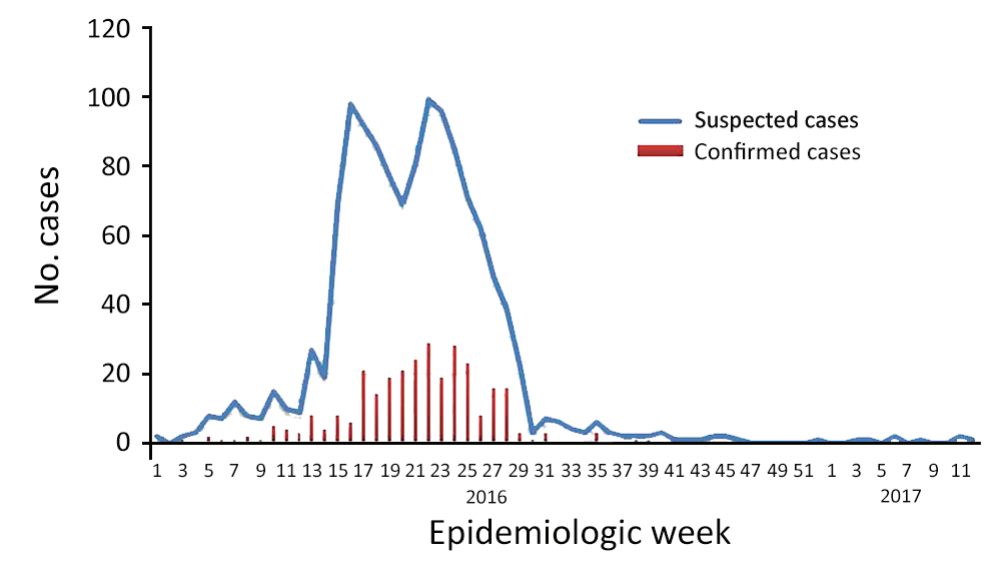
Epidemic curve of Zika virus infections among pregnant women by epidemiologic week, Dominican Republic, January 2016–April 2017.

We recorded 1,282 pregnant women with suspected Zika virus infection during the study period. Their median age was 26 (IQR 21–30) years; 16% (201/1,282) were <19 years of age (range 12–19 years). Most (91%) infections occurred during April–September 2016 (Figure 1), and a substantial proportion (28%) of suspected cases were diagnosed during the first trimester of pregnancy.

**Figure 2:**
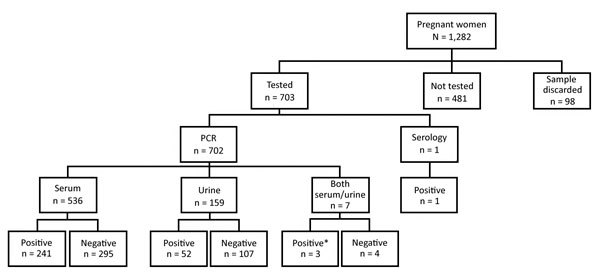
Flowchart of laboratory testing for Zika virus during the Zika epidemic in the Dominican Republic, 2016–2017. *Indicates that either sample tested positive.

Of 799 women we tested for Zika virus by PCR, 296 (37%) infections were confirmed (Table 1; Figure 2). We did not perform testing in 481 women, and we discarded 98 samples because of problems during collection or transportation. One woman had positive serologic results for IgM, meeting the definition of a probable case. We found no significant differences in the distribution of the groups, except for age, in which we observed a higher proportion of women >30 years of age testing PCR negative.

**Table 1:** Characteristics of pregnant women with suspected Zika virus infection, Dominican Republic, 2016–2017*

Characteristic	No. (%) women, n = 1,282	No. (%) PCR positive, n = 296	No. (%) PCR negative, n = 406	No. (%) not tested or sample discarded, n = 580	p value†
Age, y					
>30	308 (24.0)	60 (20.3)	113 (27.8)	135 (23.3)	0.02
<30	974 (76.0)	236 (79.7)	293 (72.2)	445 (76.7)	
Age distribution, y					
12–19	201(15.7)	45 (15.2)	59 (14.5)	97 (16.7)	0.23
20–29	704 (54.9)	173 (58.4)	211 (52.0)	320 (55.2)	
30–39	357 (27.8)	73 (24.7)	126 (31.0)	158 (27.2)	
>40	20 (1.6)	5 (1.7)	10 (2.5)	5 (0.9)	
Insurance status					
Yes	762 (59.5)	168 (56.8)	225 (55.4)	369 (63.6)	0.83
No	362 (28.2)	87 (29.4)	118 (29.1)	157 (27.1)	
Unknown	158 (12.3)	41 (13.8)	63 (15.5)	54 (9.3)	
Region of residence					
Greater Santo Domingo‡	536 (41.8)	106 (35.8)	165 (40.6)	265 (45.7)	0.19
Other	746 (58.2)	190 (64.2)	241 (59.4)	315 (54.3)	
Country of origin					
Dominican Republic	1273 (99.3)	293 (99.0)	402 (99.0)	578 (99.7)	1.00
Haiti	7 (0.5)	2 (0.7)	3 (0.7)	2 (0.3)	
Other	2 (0.2)	1 (0.3)	1 (0.3)		
Care setting					
Ambulatory/at home	1,098 (85.6)	263 (88.8)	349 (86.0)	486 (83.8)	0.26
Hospital	174 (13.6)	33 (11.2)	54 (13.3)	87 (15.0)	
Unknown (includes referred)	10 (0.8)	0	3 (0.7)	7 (1.2)	
Complications					
None	128 (10.0)	24 (8.1)	41 (10.1)	63 (10.8)	0.41
Difficulty breathing	3 (0.2)	0	2 (0.5)	1 (0.2)	
Unknown	1151 (89.8)	272 (91.9)	363 (89.4)	516 (89.0)	
Diagnosis					
Confirmed	296 (23.1)				
Suspected	983 (76.9)				
Missing	3				
Time of suspected Zika infection					
≤12 wk gestation	364 (28.4)	106 (35.8)	152 (37.4)	106 (18.3)	0.66
>12 wk gestation	918 (71.6)	190 (64.2)	254 (62.6)	474 (81.7)	
Condition of newborn at birth					
IUFD/miscarriage	70 (8.9)	26 (13.1)	22 (8.8)	22 (6.5)	0.15
Live birth	718 (91.1)	173 (86.9)	228 (91.2)	317 (93.5)	
Missing	494	97	156	241	
Premature birth, live born only, n = 718					
Yes	78 (10.9)	21 (12.1)	24 (10.5)	33 (10.5)	0.61
No	638 (89.1)	152 (87.9)	204 (89.5)	282 (89.5)	
Missing	2			2	
Birthweight, live born only, n = 718					
<2,500 g	107 (15.4)	27 (16.2)	36 (16.6)	44 (14.2)	0.91
≥2,500 g	588 (84.6)	140 (83.8)	181 (83.4)	267 (85.8)	
Missing	23	6	11	6	
Microcephaly§	3	0	1	2	

*IUFD, intrauterine fetal demise. †χ^2^ p value for the comparison of confirmed PCR positive with PCR negative. ‡Includes residents of Monte Plata Province. §Defined as head circumference <2 SD below the mean, adjusted for gestational age and sex, 24 h after birth.

**Figure 3:**
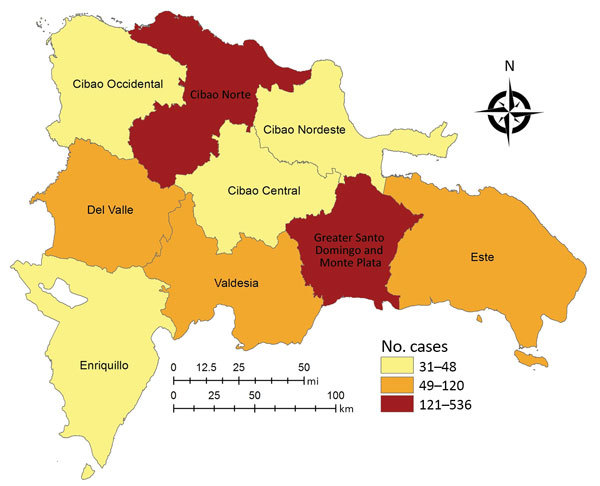
Distribution of suspected Zika virus infection in pregnant women in the Dominican Republic, by region, 2016–2017.

Most women (99.3%) were from the Dominican Republic; the remainder were from Haiti (n = 7), the United States (n = 1), and the Democratic Republic of the Congo (n = 1). Almost half the women (42%) lived in Greater Santo Domingo (Figure 3). Most (86%) received outpatient treatment, and 14% required hospitalization for severe Zika-related symptoms at the time of acute illness. Thirty-two women (4%) required a cesarean section.

Clinical data were available for 911 (71%) women. The most commonly reported symptoms were rash and arthritis/arthralgia (Table 2). The groups were homogeneous except for the finding of a higher occurrence of conjunctivitis among women who tested negative by PCR.

**Table 2:** Symptoms by PCR test result in pregnant women with suspected Zika virus infection with available clinical data, Dominican Republic, 2016–2017

Symptom	No. (%) women, n = 911	No. (%) PCR positive, n = 225	No. (%) PCR negative, n = 290	No. (%) not tested or sample discarded, n = 396	p value*
Rash					
Yes	799 (87.7)	205 (91.1)	252 (86.9)	342 (86.4)	0.13
No	112 (12.3)	20 (8.9)	38 (13.1)	54 (13.6)	
Fever					
Yes	338 (37.1)	88 (39.1)	120 (41.4)	130 (32.8)	0.60
No	573 (62.9)	137 (60.9)	170 (58.6)	266 (67.2)	
Headache					
Yes	317 (34.8)	74 (32.9)	99 (34.1)	144 (36.4)	0.77
No	594 (65.2)	151 (67.1)	191 (65.9)	252 (63.6)	
Myalgia					
Yes	189 (20.8)	46 (20.4)	64 (22.1)	79 (20.0)	0.66
No	722 (79.2)	179 (79.6)	226 (77.9)	317 (80.0)	
Arthralgia/arthritis					
Yes	449 (49.3)	108 (48.0)	147 (50.7)	194 (49.0)	0.54
No	462 (50.7)	117 (52.0)	143 (49.3)	202 (51.0)	
Conjunctivitis					
Yes	292 (32.1)	65 (28.9)	110 (37.9)	117 (29.6)	0.03
No	619 (67.9)	160 (71.1)	180 (62.1)	279 (70.4)	
Nausea/vomiting					
Yes	57 (6.3)	16 (7.1)	23 (7.9)	18 (4.6)	0.73
No	854 (93.7)	209 (92.9)	267 (92.1)	378 (95.4)	
Cough					
Yes	8 (0.9)	1 (0.4)	6 (2.1)	1 (0.2)	0.14
No	903 (99.1)	224 (99.6)	284 (97.9)	395 (99.8)	

*χ^2^ and Fisher exact test p value for the comparison of PCR positive with PCR negative.

### Pregnancy Outcomes

Data on the outcome of pregnancy were available for 788 (61%) women. A total of 718 (91%) were live births, 24 (3%) ended in IUFD, and 46 (6%) ended in miscarriage. Among live births, the median birthweight was 3,175 g (IQR 2,722–3,402 g), and 11% (78/718) were born prematurely. In most premature births (72%), the mother acquired Zika virus infection in the second or third trimester.

### Infant Outcomes

 A total of 14 congenital malformations were reported through SINAVE: suspected microcephaly (n = 9), anencephaly (n = 1), hydrocephaly (n = 1), palate fissure (n = 1), “small heart” (n = 1), and other or unspecified (n = 1). In 6 of these cases, the mother was Zika positive by PCR (suspected microcephaly in 5 and 1 “small heart”). Only 3 cases of microcephaly met the case definition of head circumference <2 SD below the mean (attack rate among all pregnant women = 0.2%; attack rate among women for whom pregnancy outcome was determined = 0.4%). One of the 3 mothers, 34 years of age, was symptomatic with Zika infection in the first month of pregnancy. The second mother, 17 years of age, was symptomatic early in the third trimester (29.5 wks) and required hospitalization for Zika-related illness. The third mother, 16 years of age, became symptomatic in the second trimester (21 wks).

### Factors Associated with Birth Outcomes

Multivariable analysis included the 788 women with a known pregnancy outcome. In crude analysis (Table 3), the odds of fetal loss were increased among women with confirmed Zika diagnosis, those who were infected in the first trimester, and those who had fever at the time of infection. The odds of premature live birth were also increased for women with fever at time of infection. In multivariable adjusted analyses, women infected with Zika virus during the first trimester were more likely to have an early fetal loss (adjusted odds ratio 5.9, 95% CI 3.5–10.0) than term birth, controlling for maternal age and symptom of fever at infection. We found no association between maternal age or timing of Zika infection in pregnancy and premature birth compared with term birth, although experiencing fever during the infection remained associated with increased odds of premature birth (adjusted odds ratio 1.65, 95% CI 1.03–2.65).

**Table 3:** Bivariate and multivariate analyses of independent variables with multinomial pregnancy outcome for women in Zika virus epidemic, Dominican Republic, 2016–2017*

Characteristic	Birth outcome			p value by χ^2^ test	Bivariate crude OR (95% CI)			Multivariate adjusted OR (95% CI), n = 786	
	Miscarriage or IUFD, n = 70	Premature live birth, n = 78	Term live birth, n = 638		Fetal loss vs. term live birth	Premature vs. term live birth		Fetal loss vs. term live birth	Premature vs. term live birth
Maternal age, y									
>30	24 (34.3)	20 (25.6)	162 (25.4)	0.27	1.53 (0.91–2.59)	1.01 (0.59–1.74)		1.48 (0.86–2.56)	1.02 (0.60–1.76)
<30	46 (65.7)	58 (74.4)	476 (74.6)	NA	NA	NA		NA	NA
Residence in Greater Santo Domingo†									
Yes	27 (38.6)	39 (50.0)	262 (41.1)	0.27	0.90 (0.54–1.50)	1.44 (0.90–2.30)		NA	NA
No	43 (61.4)	39 (50.0)	376 (58.9)	NA	NA	NA		NA	NA
Diagnosis									
Confirmed	26 (37.1)	21 (26.9)	152 (23.9)	0.05	1.88 (1.12–3.16)	1.18 (0.69–2.00)		NA	NA
Suspected	44 (62.9)	57 (73.1)	485 (76.1)	NA	NA	NA		NA	NA
Timing of suspected Zika infection, wks gestation									
<12	46 (65.7)	22 (28.2)	156 (24.4)	<0.0001	5.92 (3.50–10.0)	1.21 (0.72–2.05)		5.92 (3.49–10.0)	1.22 (0.72–2.07)
>12	24 (34.3)	56 (71.8)	482 (75.6)	NA	NA	NA		NA	NA
Symptoms‡									
Rash	60 (85.7)	70 (89.7)	568 (89.0)	0.68	0.74 (0.36–1.51)	1.08 (0.50–2.33)		NA	NA
No rash	10 (14.3)	8 (10.3)	70 (11.0)	NA	NA	NA		NA	NA
Fever	31 (44.3)	36 (46.2)	218 (34.2)	0.04	1.53 (0.93–2.52)	1.65 (1.03–2.65)		1.63 (0.97–2.75)	1.66 (1.03–2.66)
No fever	39 (55.7)	42 (53.8)	420 (65.8)	NA	NA	NA		NA	NA

*IUFD, intrauterine fetal death; NA, not applicable; OR, odds ratio. †Includes residents of Monte Plata Province. ‡No significant difference for other symptoms.

## Discussion

This report describes a cohort of suspected and confirmed Zika virus–infected pregnant women in the Dominican Republic during 2016–2017. Our analysis demonstrated substantial maternal and infant illness during the epidemic. Almost 10% of pregnancies with a known outcome resulted in early fetal loss, and there were 3 cases of microcephaly. First-trimester prenatal exposure was highly associated with fetal loss, and fever was associated with prematurity.

The frequency of symptoms in women of this cohort was consistent with other studies. Rash and arthralgia were also the most prevalent symptoms in a cohort of pregnant women with confirmed Zika virus infection in Rio de Janeiro, Brazil (*27*). The type of exanthem most commonly described in our study and others was maculopapular rash (*28*). Similarly, rash and arthralgia were the most common signs during the Zika virus outbreak on Yap Island (*14*). 

Consistent with our findings, fever was present in <50% of confirmed cases in various studies (*28*,*29*). We observed a link between the presence of fever and prematurity. Fever in the mother may reflect a more inflammatory infection or may be unrelated to Zika virus and result from co-infection with other pathogens. Fever can also be associated with other conditions, such as premature rupture of membranes resulting from genital tract infection. The limited available information on women’s prenatal and peripartum care precludes drawing sound conclusions on this observation. Data from ongoing prospective cohort studies may help further elucidate this finding. 

Advanced maternal age was not associated with prematurity or fetal loss in our study, a finding that is not surprising in this cohort because most older women (92%) were 30–35 years of age, a range below the usual threshold for pregnancy complications. Zika virus infection can lead to birth defects and pregnancy complications even when the mother is asymptomatic, but this report contains only surveillance data. Women had to be symptomatic to trigger reporting to the MoH, and thus, it is not possible to estimate the burden of disease in asymptomatic women and their infants. The MoH prioritized and directed its limited resources to testing of symptomatic pregnant women during this relatively sudden and explosive epidemic.

Fetal loss has been documented in an experimental animal model of marmoset monkeys. The inoculation of Zika virus into pregnant females caused prolonged fetal and placental viral replication and a maternal associated host response and increased activity of proinflammatory cytokines (*30*). The rate of fetal loss in Zika virus–infected women is estimated at 3%, and rate of birth defects is ≈4%–8%, depending on the trimester of infection (*31*). In our study, women reported as symptomatic during the first trimester of pregnancy had 4 times greater odds of fetal loss that those with later symptoms, after controlling for maternal age. Similar pregnancy complications have been documented in other recent cohorts. In Brazil, 13 (7%) fetal losses and 4 cases of microcephaly were reported among 186 women with known pregnancy outcome. Cesarean sections were more prevalent in Brazil compared with our cohort in the Dominican Republic (81% vs. 4%) (*27*). The proportion of pregnancy losses was 11% (47/442) among women with possible Zika infection included in the US Zika Pregnancy Registry (*32*). In our cohort, 11% of births were premature, a proportion that is higher than the reported national average of 8 per 100 live births (*33*). IUFD was 3% of deliveries, which is higher than the reported national average of 1.1% (*34*)

This type of analysis of public health surveillance data has inherent limitations. First, not all women were tested for Zika virus, and their diagnosis relied on clinical reports. It is plausible that some of the suspected cases were caused by dengue virus, which is the most common arboviral illness in the country, or another infection (syphilis, toxoplasmosis, rubella, cytomegalovirus, and herpes simplex virus, the STORCH infections). However, many distinguishing clinical and laboratory features between dengue virus and Zika virus are familiar to clinicians adept at diagnosing dengue virus infection. The fact that a higher proportion of women who tested PCR negative had conjunctivitis is reassuring, given its presence helps to differentiate dengue from Zika. Second, our assessment of birth defects is limited to visible abnormalities, such as microcephaly, in live births. Birth defects were not reported on fetal losses, and there were no radiographic, ophthalmologic, or audiologic assessments to ascertain inconspicuous birth defects. Most women delivered at public hospitals and clinics with limited resources. These centers are not equipped to evaluate brain radiographic abnormalities associated with Zika virus infection. Third, clinical data are missing for almost one third of the women and pregnancy outcome is not known in 39% of cases. Fourth, true disease burden in pregnancy is underestimated because we have no data for asymptomatic women not captured by passive surveillance.

The main strength of this study is that it includes a large group of pregnant women with suspected Zika virus infection in the Caribbean region. We used the Dominican Republic government’s main reporting platform to analyze multicenter and countrywide population-level data. Our finding of increased likelihood of miscarriage and IUFD in a large population-based sample strengthens the evidence for a broad range of adverse pregnancy outcomes, building upon case reports and countrywide evaluations.

In conclusion, we documented substantial illnesses of pregnant women and their children stemming from the 2016–2017 Zika virus outbreak in the Dominican Republic. Our analysis highlights gaps in our epidemiologic understanding of the course of the Zika virus epidemic and affected populations (e.g., data not uniformly collected). Accordingly, we need to strengthen passive surveillance, implement sentinel active surveillance, and improve the timeliness and reliability of in-country diagnostic testing. The results of lessons learned about the severity of Zika and breadth of adverse outcomes and the role of surveillance in detecting and preventing adverse outcomes need to be put in place before the next outbreak.
